# Effect of Extrusion Cooking on Bioactive Compounds in Encapsulated Red Cactus Pear Powder

**DOI:** 10.3390/molecules20058875

**Published:** 2015-05-18

**Authors:** Martha G. Ruiz-Gutiérrez, Carlos A. Amaya-Guerra, Armando Quintero-Ramos, Esther Pérez-Carrillo, Teresita de J. Ruiz-Anchondo, Juan G. Báez-González, Carmen O. Meléndez-Pizarro

**Affiliations:** 1 Departamento de Investigación y Posgrado, Facultad de Ciencias Químicas, Universidad Autónoma de Chihuahua, Circuito Universitario s/n Campus Universitario 2, Chihuahua 31125, Mexico; E-Mails: martha.ruiz.mr@gmail.com (M.G.R.-G.); carmenoralia@yahoo.com (C.O.M.-P.).; 2 Departamento de Investigación y Posgrado, Facultad de Ciencias Biológicas, Universidad Autónoma de Nuevo León, Ciudad Universitaria, San Nicolás de los Garza 66450, Mexico; E-Mails: numisamaya@hotmail.com (C.A.A.-G.); juan.baezgn@uanl.edu.mx (J.G.B.-G.); 3 Centro de Biotecnología-FEMSA, Escuela de Ingenería y Ciencias, Tecnológico de Monterrey, Av. Eugenio Garza Sada 2501 Sur, Monterrey 64849, Mexico; E-Mail: perez.carrillo@itesm.mx; 4 Facultad de Ciencias Agrotecnológicas, Universidad Autónoma de Chihuahua, Circuito Universitario s/n Campus Universitario 1, Chihuahua 31310, Mexico; E-Mail: teresita_anchondo@hotmail.com

**Keywords:** red cactus pear powder, extrusion cooking, betalains, bioactive compounds

## Abstract

Red cactus pear has significant antioxidant activity and potential as a colorant in food, due to the presence of betalains. However, the betalains are highly thermolabile, and their application in thermal process, as extrusion cooking, should be evaluated. The aim of this study was to evaluate the effect of extrusion conditions on the chemical components of red cactus pear encapsulated powder. Cornstarch and encapsulated powder (2.5% w/w) were mixed and processed by extrusion at different barrel temperatures (80, 100, 120, 140 °C) and screw speeds (225, 275, 325 rpm) using a twin-screw extruder. Mean residence time (*tr_m_*), color (L*, a*, b*), antioxidant activity, total polyphenol, betacyanin, and betaxanthin contents were determined on extrudates, and pigment degradation reaction rate constants (*k*) and activation energies (*E_a_*) were calculated. Increases in barrel temperature and screw speed decreased the *tr_m_*, and this was associated with better retentions of antioxidant activity, total polyphenol, betalain contents. The betacyanins *k* values ranged the −0.0188 to −0.0206/s and for betaxanthins ranged of −0.0122 to −0.0167/s, while *E_a_* values were 1.5888 to 6.1815 kJ/mol, respectively. The bioactive compounds retention suggests that encapsulated powder can be used as pigments and to provide antioxidant properties to extruded products.

## 1. Introduction

There is a growing interest to include in diets foods that are manufactured with natural functional ingredients. In Mexico, there are many foods that can be considered as functional products; cactus pear is one of these. The fruit is fleshy and appears in various colors, *i.e.*, white, yellow, and red-purple [1]. In particular, the red cactus pear (*Opuntia ficus indica*) has a red-purple pulp, which is due to the presence of betalains. These pigments are betalamic acid derivatives and are divided into two structural groups: the red-violet betacyanins, e.g., betanin, neobetanin, and isobetanin, and the yellow-orange betaxanthins, e.g., indicaxanthin and vulgaxanthin I, II, and IV [2]. Recent studies have shown that betalains are antioxidants and are able to delay the effects of oxidative damage [3], among other health benefits [4,5]. In addition, the betalains are a potential source of natural dyes that can be used in the food industry. However, the stability of these pigments is affected by factors such as temperature, pH, water activity, and presence of oxygen, light, and metals. The temperature is the main factor that affects the color stability and biological activity of the pigments [6,7,8], therefore, the degradation of red cactus pear pigments during processing through conventional methods or new technologies should be evaluated. 

Extrusion cooking is a technology that is widely used for the development of new products. During this process the feed is transported, mixed and cooked at a high temperature for a short time to obtain expanded products, provoking some physical and chemical changes in the feed material [9]. The major changes that may occur in the feed material during the process are starch gelatinization, protein denaturation, complex formation (amylose-lipid), and degradation of heat sensitive components such as vitamins, antioxidants, and pigments [10,11,12]. These changes depend upon process conditions, e.g., types of extruder, feed material, material moisture content, barrel temperature, and screw speed, among other factors [13]. These operation factors are related with residence time distribution (*RTD*), which is a useful tool to study physicochemical changes in feed materials. Additionally, *RTD* has been used to scale-up operations [14]. Recent extrusion process studies had been focused in the development of functional products using legumes [15], cereals [16], fruits [17,18,19], fruit extracts [20], and vegetables [21]. The results showed that the extrusion cooking process might cause a decrease in the concentration by structural changes of compounds with antioxidant activity such as carotenoids [22,23], anthocyanins [10,20,23,24], and polyphenols [22,23,25], attributed mainly to the high process temperature. Some authors [10] studied the use of fruit powders (blueberry, grape, and raspberry) to pigment extruded cereals. They found that although the cereals had a good color, anthocyanin and phenolic contents decreased up to 10-fold. Other studies reported the use of encapsulated powders in the extrusion process to enhance the retention of these components. The use of encapsulated d-limonene in the extrusion cooking process has been reported [26], finding a high retention for d-limonene due to the formation of inclusion complexes with starch, obtaining products with lesser components degradation. However, the behavior of the bioactive compounds of encapsulated red cactus pear, e.g., betalains, processed by extrusion cooking has not been investigated, therefore their stability on extruded products should be evaluated due to that the pigments represent a dye alternative and source of antioxidants in the development of food products. The aim of this study was to evaluate the effect of barrel temperature and screw speed during the extrusion cooking process on bioactive compounds of encapsulated red cactus pear powder.

## 2. Results and Discussion

### 2.1. Residence Time Distribution (RTD)

The *RTD* of the material in the extruder under different barrel temperature and screw speed conditions is characterized by *tr_m_*, which is shown in Table 1. The *tr_m_* values obtained for the different extrusion conditions were between 36.62 and 60.13 s. These values represent the average time that the feed material remains in the extruder and time that it is exposed to high temperature and shear stress. The residence time is associated with changes that occur in the feed material and that result in complex formation or degradation of the components of interest.

**Table 1:** Mean residence time ^+^ of extrudates at different process conditions using red cactus pear encapsulated.

Screw Speed (rpm)	Temperature (°C)	*tr_m_* (s)
225	80	60.13 ± 2.95 ^a^
	100	56.20 ± 2.28 ^a^
	120	56.18 ± 0.09 ^a^
	140	47.72 ± 1.43 ^b^
275	80	47.82 ± 0.13 ^b^
	100	48.59 ± 1.67 ^b^
	120	48.74 ± 0.66 ^b^
	140	46.75 ± 2.81 ^bc^
325	80	40.84 ± 0.82 ^cde^
	100	43.12 ± 0.76 ^bcd^
	120	36.62 ± 1.63 ^e^
	140	37.53 ± 0.98 ^de^

^+^ Mean ± standard deviation; Values with different letter by column indicate significant difference (*p* < 0.05) by Tukey’s test.

**Figure 1:**
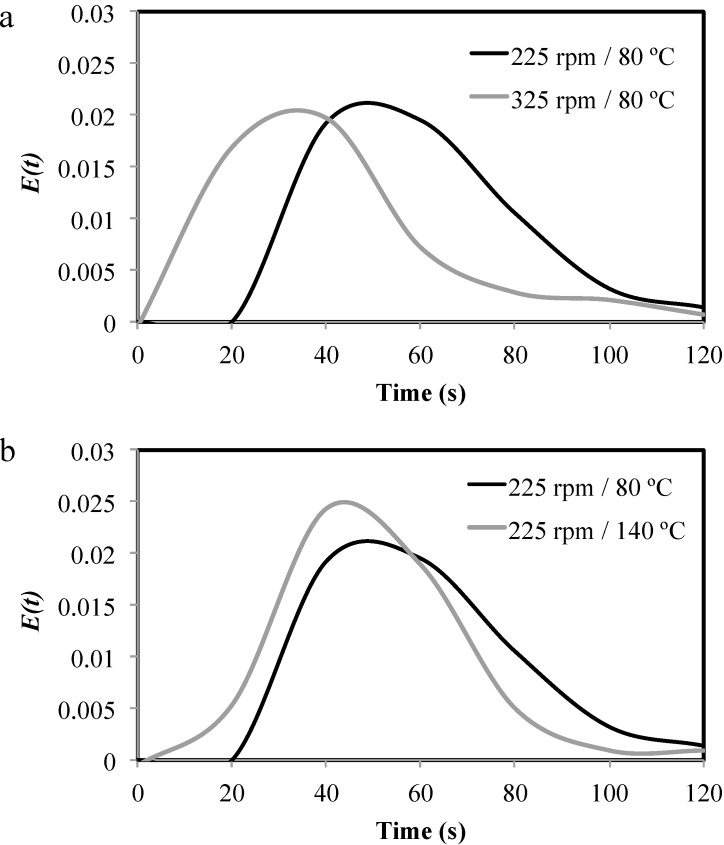
Residence time distribution (*RTD*) at different conditions used in the extrusion cooking process: (**a**) screw speed effect; (**b**) temperature effect.

*tr_m_* was significantly affected (*p* < 0.05) by barrel temperature, screw speed, and the interaction of both factors (Table 1). The data shows that increases in the screw speed at the same temperature, the material *tr_m_* decreased (Figure 1a). This may be due to increased heat dissipation, higher shear stress, and/or better mixing of the material during process [11,27], resulting in shorter residence times as the speed of the extruding screw increases. Similar findings have been reported by others authors [28,29]. Moreover, increasing the temperature in the extrusion process resulted in a decrease in *tr_m_* (Figure 1b). This can be attributed to the reduction in the viscosity of the material in transit as the temperature increases, which produces a more fluid material through the barrel and screw [11,27]. The same effect of temperature on *tr_m_* was reported before [27,28,30].

### 2.2. Extrudate analyses

#### 2.2.1. Moisture and Color Parameters

The extrudates had mean moisture content of 9.26% ± 0.3%. The color analysis results of the extrudates are shown in Table 2. The three parameters measured, L* (lightness), a* (greenness-redness), and b* (blueness-yellowness), were significantly affected (*p* < 0.05) by the process temperature and screw speed, as well as by the interaction of both factors. 

Low L* values were obtained when the extrusion was carried out at the highest temperature (140 °C) at 225 and 325 rpm. This result may indicate the formation of some Maillard products, which cause darkening of the extrudates. Others authors observed the same tendency [31]. This behavior can also be related to the *tr_m_* obtained (Table 1), as the increase in temperature causes a decrease in the mean residence time.

**Table 2:** Color analyses ^+^ of extrudates at different process conditions using red cactus pear encapsulated.

Screw Speed (rpm)	Temperature (°C)	L*	a*	b*
225	80	82.21 ± 3.73 ^a^	23.29 ± 0.94 ^bc^	4.34 ± 0.10 ^f^
	100	83.11 ± 2.17 ^a^	23.30 ± 1.54 ^bc^	5.42 ± 0.43 ^def^
	120	82.43 ± 4.52 ^a^	22.52 ± 1.48 ^cd^	6.12 ± 0.91 ^cd^
	140	75.67 ± 2.37 ^bc^	25.27 ± 0.91 ^a^	9.74 ± 0.47 ^a^
275	80	84.07 ± 2.07 ^a^	20.71 ± 0.44 ^def^	5.02 ± 0.11 ^f^
	100	85.37 ± 2.09 ^a^	20.44 ± 0.70 ^ef^	5.68 ± 0.15 ^def^
	120	81.85 ± 1.47 ^a^	21.47 ± 0.90 ^cde^	6.89 ± 0.33 ^c^
	140	80.81 ± 4.49 ^ab^	19.44 ± 1.25 ^f^	8.30 ± 0.18 ^b^
325	80	83.65 ± 2.04 ^a^	19.84 ± 0.54 ^ef^	5.21 ± 0.17 ^f^
	100	83.65 ± 2.12 ^a^	19.66 ± 0.82 ^ef^	5.87 ± 0.36 ^de^
	120	85.44 ± 3.28 ^a^	18.94 ± 0.91 ^f^	7.81 ± 0.45 ^b^
	140	72.52 ± 3.18 ^c^	24.78 ± 0.79 ^ab^	10.11 ± 0.43 ^a^

^+^ Mean ± standard deviation; Values with different letters by column indicate significant difference (*p* < 0.05) by Tukey’s test.

The parameter a*, which represents the change from green to red, showed that the obtained extrudates tended to be red, because the measured values were positive (Table 2). The highest values were obtained at screw speeds of 225 and 325 rpm and at the highest process temperature (140 °C). This result may be due to high color retention of components that provide red-purple colors; high retention or reduced degradation may be due to the applied conditions, *i.e.*, the *tr_m_* of the material in the extruder was the shortest (Table 1). In addition, the tendency to red color can be attributed to the increase in the formation of Maillard products; the formation of such products increases as the process temperature increases [32].

With regard to the color parameter b*, which represents the change from blue to yellow, only positive values were obtained, indicating a tendency to yellow (Table 2). An increase in the process temperature at same speed screw, resulted in a marked color change to yellow; the highest b* values were measured in extrudates obtained at the highest process temperature (140 °C). These results can be due to the formation of Maillard products by increases in barrel temperature [32].

#### 2.2.2. Total Polyphenol Content

**Figure 2:**
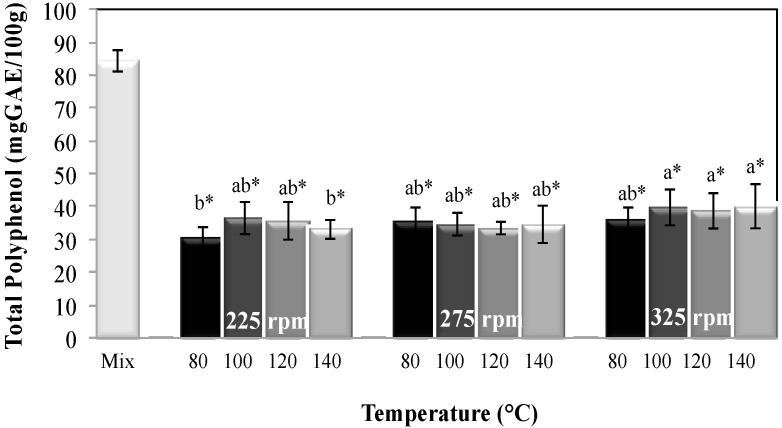
Total polyphenol content of the mixture and extrudates obtained at different extrusion cooking process conditions. Columns with different letters are significantly different; * indicates a significant difference between raw mixture and extrudate (*p* < 0.05) by Tukey’s test.

The total polyphenol content is shown in Figure 2. This parameter was significantly affected (*p* < 0.05) by the process temperature and screw speed. The highest values of total polyphenol content were obtained at the highest screw speed used (325 rpm); this can be attributed at the decreasing of the residence time reached by screw speed increase. Low values were obtained at the lowest screw speed used (225 rpm), which can be due to the longer exposure time (Table 1) of polyphenolics compounds to the severe conditions of temperature and screw speed in the extrusion process. 

Furthermore, a significant reduction in the total polyphenol content was observed, in comparison with the initial content of these components in the raw mixture. The retention values calculated for the different extrusion conditions were in the range of 36% to 47%. These results indicate that the extrusion process causes a decrease of the phenolic compounds; temperatures over 80 °C may destroy or alter their nature [33]. The extrusion cooking process will lead to a decrease of phenolics because of either phenolic compound decomposition or alterations in their molecular structure caused by the high extrusion temperature [34]. In specific the decarboxylation of free phenolic acids during the extrusion process [35], as well as the formation of insoluble complexes with food components like proteins [35]. In addition, high temperature together with feed high moisture content, can promote polymerization of phenols; reducing the extraction capacity, chemical reactivity [16,23,25] and quantification. The decrease in phenolic compounds has been observed in the extrusion cooking [23,25,34,35,36], e.g., in beans reductions between 10% to 70% was reported [15] and in amaranth an 80.3% reduction was reported [37].

#### 2.2.3. Antioxidant Activity

**Figure 3:**
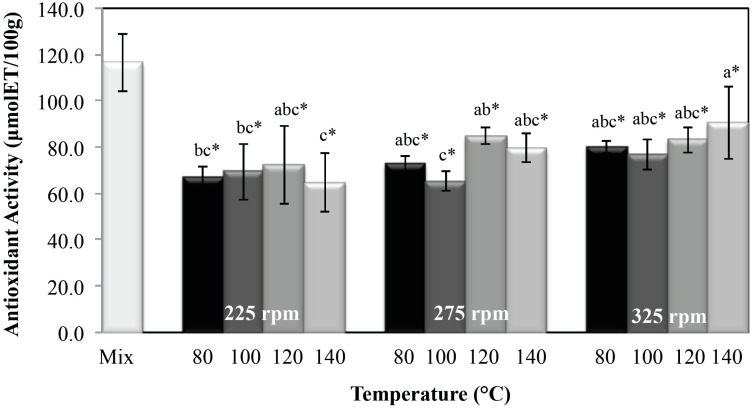
Antioxidant activity of the mixture and extrudates obtained at different extrusion cooking process conditions. Columns with different letters are significantly different; * indicates a significant difference between raw mixture and extrudate, (*p* < 0.05) by Tukey’s test.

The antioxidant activity of the extrudates showed the same behavior as the observed for the total polyphenol content. Figure 3 shows that the antioxidant activity was significantly affected (*p* < 0.05) by barrel temperature and screw speed. Antioxidant activity increased as screw speed and extrusion temperature increased. The highest antioxidant activity values were found in extrudates processed at the highest screw speed (325 rpm) and highest temperature (140 °C). This was associated with a low *trm* of the material in the extruder (Table 1), which indicates a short exposure time of the components could lead to a less degradation of betalains and polyphenols resulting in higher antioxidant activity of the extrudates. 

However, the antioxidant activity values of all extrudates were lower than the antioxidant activity determined in the raw mixture. The retention of antioxidant activity at the different processing conditions was calculated between 55% and 77%. The retention values obtained in the present study are within the range reported by others [15]. The decrease in antioxidant activity may be related to the degradation of polyphenols and pigments during the process, due to the fact that the antioxidant potency of these components arises from their structure, specifically from their ability to donate hydrogen atoms from hydroxyl groups to free radicals [36]. The extrusion cooking process will lead to a decrease of compound due to alterations in their molecular structure caused by the high temperature [34], thereby reducing antioxidant activity [16,34,35]. 

#### 2.2.4. Betalain Content

**Figure 4:**
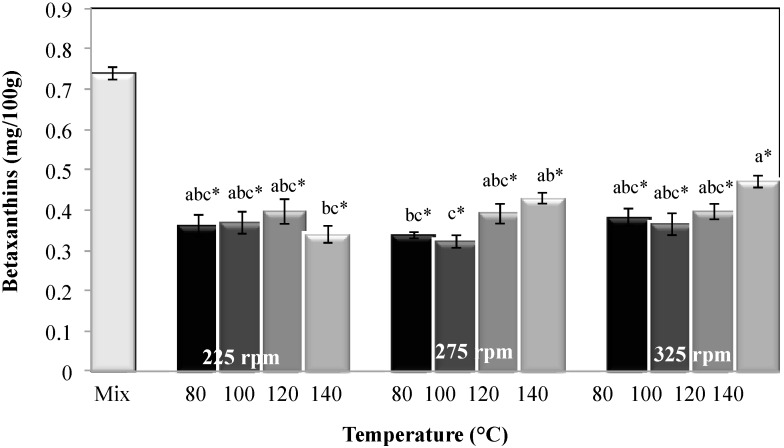
Betaxanthin content in the raw mixture and extrudates obtained at different extrusion cooking process conditions. Columns with different letters are significantly different; * indicates a significant difference between raw mixture and extrudate (*p* < 0.05) by Tukey’s test.

Betalain analyses showed that the betaxanthin content (Figure 4) was significantly affected (*p* < 0.05) by process temperature, screw speed, and the interaction of these factors. The lowest betaxanthin contents were found under the following conditions: 225 rpm and 140 °C and 275 rpm at 80 and 100 °C. This is related to the *tr_m_* of the material in the extruder, *i.e.*, a larger *tr_m_* results in a longer exposure time of the components to a high temperature during the process, which causes increases in the degradation of pigments because they are sensitive to temperature [2]. Moreover, a marked decrease in the betaxanthin content was due to the extrusion cooking process. The retention values ranged between 46% and 63.5%, with respect to the amount of feed raw mixture. 

**Figure 5:**
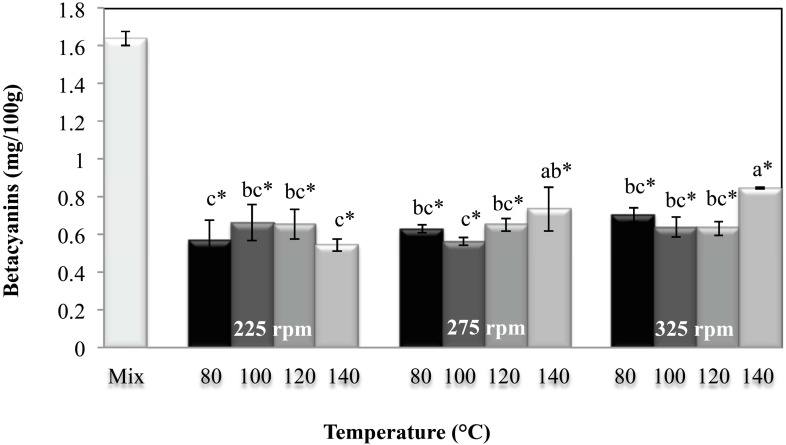
Betacyanin content in the raw mixture and extrudates obtained at different extrusion cooking process conditions. Columns with different letters are significantly different; * indicates a significant difference between raw mixture and extrudate (*p* < 0.05) by Tukey’s test.

The betacyanin content was significantly affected (*p* < 0.05) by the screw speed and the interaction of screw speed and temperature, as shown in Figure 5. 

The highest betacyanin contents were obtained at high screw speeds (275 and 325 rpm) and at highest temperature (140 °C). This effect is due to the *tr_m_*, of the material in the extruder, which were the lowest under these conditions (Table 1). This result indicates that low extrusion temperature and screw speed result in further degradation of heat-sensitive components such as pigments. Other studies have also reported that an increase in the barrel temperature causes an increase in the retention of components sensitive to temperature such as certain vitamins. This is possibly due to the reduction in shear stress as result from the reduction in melt viscosity at high extrusion temperatures [37]. 

In addition, pigment loss caused by the extrusion cooking process was observed. The calculated retention values were in the range of 33% to 51%, indicating that more than half of these components were lost during the process. The use of temperature in different process causes a degradation of betalains [38]. During thermal treatment the main pigment in red cactus pear (betanin) can be degraded by isomerization, decarboxylation, or cleavage [39]. Pigment loss as antocyanins, carotenes and color loss of betalains caused by the extrusion cooking process has been reported [17,19,22,24,40]. In [40] the authors reported retention of 50% for the beetroot pigment processed by extrusion cooking, but in that study, only color retention was measured. Similar results were reported for total anthocyanins (33%–42%) during cranberry pomace extrusion cooking at high temperature (160 and 180 °C) and low screw speeds (150 and 200 rpm) [17]. Other reports have shown pigment reductions of 64% to 90% in breakfast cereals with added berry concentrates [24], and reductions of about 90% in extruded products with added cranberry, raspberry and grape fruit powders [10]. The influence of the temperature or mechanical stress caused by the extrusion conditions on the betalains has not been completely explained yet. However, has been reported that the thermal and mechanical stress during extrusion process causes degradation of pigments as β-carotene, result of the oxidation [22], which could be happening in the betalain molecules.

#### 2.2.5. Betacyanin Retention

The predominant pigments in red cactus pear are betanin and isobetanin; both pigments belong to the red-purple color betacyanins group. To determine the betacyanin retention, betanin and isobetanin were monitored during the extrusion cooking process. The retention values of both pigments at different extrusion conditions are shown in Table 3. 

**Table 3:** Betacyanins retention * of extrudates obtained using red cactus pear encapsulated.

Speed Screw (rpm)	Temperature (°C)	Betanin (%)	Isobetanin (%)
225	80	39.12 ± 1.21 ^ab^	107.36 ± 14.41 ^de^
	100	42.39 ± 2.26 ª	129.83 ± 6.85 ^cde^
	120	26.59 ± 1.84 ^c^	150.86 ± 26.04 ^bc^
	140	13.58 ± 1.35 ^e^	152.82 ± 16.62 ^bc^
275	80	41.70 ± 0.14 ^a^	98.35 ± 26.04 ^e^
	100	30.96 ± 3.84 ^c^	135.18 ± 18.29 ^bcde^
	120	24.14 ± 5.58 ^cd^	200.23 ± 20.50 ^a^
	140	10.92 ± 1.55 ^e^	132.18 ± 0.90 ^cde^
325	80	43.54 ± 1.62 ^a^	120.42 ± 2.52 ^cde^
	100	31.83 ± 2.34 ^bc^	142.63 ± 5.54 ^bcd^
	120	17.03 ± 3.43 ^de^	150.47 ± 14.41 ^bcd^
	140	15.55 ± 2.07 ^e^	166.53 ± 11.64 ^ab^

* Mean ± standard deviation; Values with different letter by column indicate significant difference (*p* <0.05) by Tukey’s test.

The retention values of betanin ranged between 10.92% and 43.54%. The concentration of this pigment was significantly affected (*p* < 0.05) by temperature, screw speed, and the interaction of both factors. It was observed that an increase in the barrel temperature caused a decrease in the betanin concentration. The highest retention values were obtained at the lowest temperature (80 °C at any speed screw and 100 °C at 225 rpm). 

The isobetanin concentration was significantly affected (*p* < 0.05) by temperature and the interaction of barrel temperature and screw speed; however, it showed a trend opposite to that observed for betanin. An increase in the isobetanin content was observed at all extrusion conditions.

The result suggests that during the extrusion process occur structural changes in the betanin by the exposure to high temperatures. The epimerization of the betanin to isobetanin may be caused by the heat [41]. The high temperature applied in extrusion can leads to isomerization and decarboxylation of betanin, producing its C_15_-stereoisomer, corresponding to isobetanin [42].

#### 2.2.6. Kinetics Parameters 

The decay constant (*−k*) and its respective determination coefficient (R*^2^*) for the different extrusion condition were calculated by kinetic analysis; they are shown in Table 4. 

**Table 4:** Effect of temperature on degradation rate constant * and activation energy of red cactus pear betalains in extrusion cooking process.

Temperature (°C)	Betalain	*k* (^1^/s)	R^2^	*E_a_* (kJ/mol)	R^2^
80	BC	−0.0206 ± 0.0005 ^a^	0.9855	1.5888 ^b^	0.9897
100		−0.0201 ± 0.0013 ^a^	0.9825		
120		−0.0194 ± 0.0010 ^a^	0.9540		
140		−0.0192 ± 0.0033 ^a^	0.9858		
80	BX	−0.0167 ± 0.0011 ^b^	0.9491	6.1815 ^a^	0.9898
100		−0.0145 ± 0.0003 ^c^	0.9869		
120		−0.0135 ± 0.0013 ^d^	0.9375		
140		−0.0122 ± 0.0002 ^d^	0.9719		

* Mean ± standard deviation; Values with different letters by column indicate significant difference (*p* < 0.05) by Tukey’s test. BC = Betacyanin; BX = Betaxanthin; *k* = Degradation rate constant; R^2^ = Determination coefficient; *E_a_* = Activation energy.

The degradation rate constants (*k*) for betacyanins were not significantly (*p* > 0.05) affected by the barrel temperatures used in the extruder. However, the *k* value tended to decrease as temperature was increased. This is due to the *tr_m_* decrease of the material in the extruder (Table 1), resulting in short exposure times of betacyanins to high temperature and shear stress during extrusion, while, the degradation rate constant of betaxanthins decreased significantly (*p* < 0.05) as the temperature applied in the extrusion cooking process increased. This can also be related to the decrease in *tr_m_* as the temperature increase (Table 1). Furthermore, the rate constants for the degradation of betacyanins were significantly (*p* < 0.05) higher than those obtained for betaxanthins.

**Figure 6:**
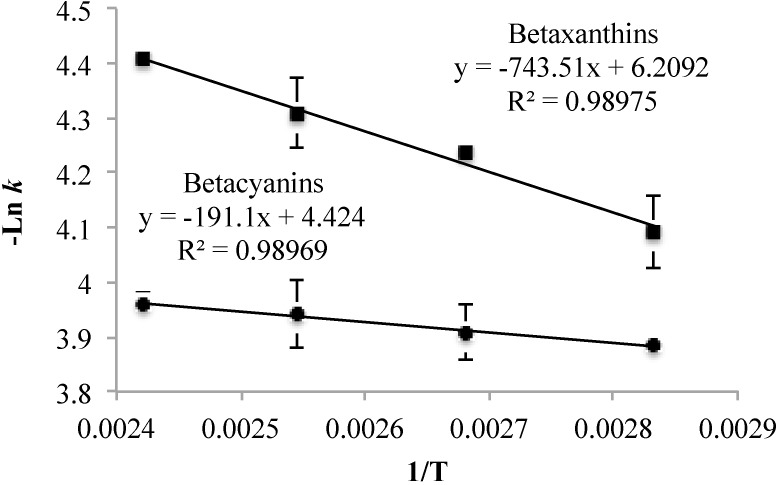
Arrhenius plot of the degradation of betaxanthins and betacyanins of red cactus pear powder used during the extrusion cooking process.

Using the *k* data, the Arrhenius plot was obtained for the degradation of betaxanthins and betacyanins and their behavior is shown in Figure 6. The activation energies for the degradation reaction were calculated and are shown in Table 4. The activation energy for the degradation of betaxanthins was greater than the degradation of betacyanins. This result indicates that betaxanthins are more susceptible to temperature increases [43] during the extrusion cooking process. 

## 3. Experimental Section 

### 3.1. Materials

Encapsulated powder of juice of red cactus pear (Puebla, Mexico) was obtained by spray drying using soluble fiber [(1→3)(1→4) β-d-glucan] (22.5%) as carrier agent. The drying process was carried out at 160 °C inlet temperature and 85 °C outlet temperature according to a reported method [44]. The encapsulated powder presented the following characteristics: moisture content of 0.924% ± 0.159%, water activity of 0.103 ± 0.02, pH of 5.53 ± 0.029, bulk density of 0.622 ± 0.096 g/mL, water solubility index of 0.711 ± 0.049, and color parameters of 43.27 ± 2.99, 38.18 ± 1.74, and −5.50 ± 0.2 for L*, a* and b*, respectively. The chemical characteristics: antioxidant activity and total polyphenol, betaxanthin, and betacyanin contents are reported in the Results and Discussion Section. The food matrix for the extrusion cooking process was native cornstarch (GPC, Muscatine, IA, USA), which was mixed with 2.5% (w/w) of encapsulated red cactus pear powder.

### 3.2. Reagents

2,2-Diphenyl-1-picrylhydrazyl (DPPH), Trolox, Folin-Ciocalteu, sodium carbonate, and gallic acid standard reagent were purchased from Sigma-Aldrich (St. Louis, MO, USA). HPLC-grade methanol, HPLC-grade water, HPLC-grade phosphoric acid and analytical grade methanol and potassium dihydrogen phosphate (KH_2_PO_4_) were from J. T. Baker (Mexico City, Mexico). Deionized water was obtained using a deionizer (Barnstead, Thermo Scientific, Waltham, MA, USA). All other reagents used were analytical grade.

### 3.3. Extrusion-Cooking Process

The mixture was fed into twin-screw corotating extruder (BCTM-30, Bühler, Uzwil, Switzerland) with a 600 mm length and L/D = 20. Die diameter was 4 mm, and the screw configuration was selected specifically to create high levels of shear [45]. The mixture was processed at fixed amount of feed water (0.22 kg water/kg dry matter), different temperatures (80, 100, 120, and 140 °C) which were controlled at the final stage of the extruding chamber by using a TT-137N water heater (Tool-temp, Sulgen, Switzerland) and at screw speeds of 225, 275, and 325 rpm. All extrudates were dried at 45 °C in an air convection oven, bagged and stored at room temperature until tested.

### 3.4. Residence Time Distribution (RTD)

Cornstarch was mixed with erythrosine (3% w/w) and introduced into the extruder using the pulse technique; effluent samples were taken from the extruder every 20 s, starting from the addition of erythrosine (time = 0). The obtained extrudates were dried at 60 °C in an air convection oven, milled, and passed through a No. 20 mesh (0.85 mm). The color parameter a* (greenness-redness) in the obtained powders was evaluated using tristimulus colorimetry. The obtained data were used to calculate the residence time distribution *E*(*t*) and *tr_m_* in the extruder [46] using the Equations (1) and (2), respectively:

Distribution function:

(1) $$E(t)=\frac{C(t)}{\int_{0}^{\infty }C(t)dt}$$

Mean residence time:

(2) $$tr_{m}=\int_{0}^{\infty }tE(t)dt$$

where *E*(*t*) is the exit age distribution function, *C*(*t*) is the tracer concentration (measured as a*) at time *t* and *dt* is the time interval between successive sampling.

### 3.5. Extrudate Analyses

#### 3.5.1. Moisture Content

The moisture content was determined using the gravimetric method 950.02 [47]. The analysis was carried out in triplicate, and results were expressed in g/100 g of extrudate.

#### 3.5.2. Color parameters

The color of the extrudate was determined using a Konica Minolta CR-400/410 colorimeter (Minolta Co., Osaka, Japan), calibrated with a tile standard with the following values: X = 94.9, y = 0.3185, and x = 0.3124. The color was expressed as L* (lightness), a* (greenness-redness), and b* (blueness-yellowness) values. The determination was carried out in triplicate. 

#### 3.5.3. Extract Preparation

To determine the polyphenol content and antioxidant activity, extracts were obtained according to the reported method [48], with some modifications. Briefly, extrudate (2.5 g) was dispersed in a mixture of methanol and deionized water (25 mL, 1:1, v/v). The dispersion was homogenized for 15 s using an Ultra-Turrax IKA T18 Basic homogenizer equipped with a S18N-19G dispersing tool (IKA Works, Inc., Wilmington, NC, USA). The mix was centrifuged at 3000× *g* for 10 min in a Centra CL3R IEC centrifuge (Thermo Electron Corporation, Waltham, MA, USA); the procedure was repeated to ensure complete extraction. The supernatants were collected and filtered through a polyethylene filter with a pore size of 0.22 μm (Millipore Corp., Bedford, MA, USA), and then stored at −20 °C until analysis.

#### 3.5.4. Total Polyphenol Content

The total polyphenol content was determined spectrophotometrically using the Folin-Ciocalteu method [49]. Mixtures were prepared by combining the extract (0.2 mL) with deionized water (1.8 mL), Folin-Ciocalteu reagent (10 mL, 10-fold diluted), and a sodium carbonate solution (8 mL, 7.5%). The mixtures were vortexed for 10 s and incubated in a water bath at 45 °C. After 15 min, the reaction was stopped by transferring them to a water/ice bath and the absorbance was measured at 765 nm on a Lambda 25 UV-Vis spectrophotometer (Perkin Elmer, Waltham, MA, USA). The measurements were performed in triplicate and the polyphenol content was calculated from a calibration curve using gallic acid as a standard. The results were expressed as mg gallic acid equivalent/g of extrudate.

#### 3.5.5. Antioxidant Activity

The antioxidant activity was determined according to the spectrophotometric method [50]. This method is based on the measurement of the absorbance of the DPPH^•^ radical. First, extract (0.1 mL) was added to a DPPH^•^ radical solution (3.9 mL, 100 μM), shaken, and stored in the dark for 3 h. The absorbance was measured at 517 nm using a Lambda 25 UV-Vis spectrophotometer (Perkin Elmer, Waltham, MA, USA). A Trolox standard calibration curve (0.08–1.28 mM) was used to calculate the antioxidant activity. Measurements were performed in triplicate and the results were expressed as mmol Trolox equivalent/100 g of extrudate.

#### 3.5.6. Betalain Extraction

Betalain pigments were extracted using a method reported [51], with some modifications. Briefly, extrudate (2.5 g) was mixed with deionized water (25 mL) and stirred for 15 s using an Ultra-Turrax IKA T18 Basic homogenizer equipped with a dispersing tool S18N-19G. The samples were centrifuged at 3600× *g* at 10 °C for 40 min in a Centra CL3R IEC centrifuge (Thermo Electron Corporation, Waltham, MA, USA); this procedure was repeated to ensure complete extraction. Supernatants were filtered through a 0.45-μm nylon filter (EMD Millipore Corp, Billerica, MA, USA). The extractions were performed in triplicate and the extracts were analyzed by photometric and high-performance liquid chromatography methods. 

#### 3.5.7. Photometric Quantification of Betalains

The betalain content was determined photometrically following a method reported [51] using a Lambda 25 UV-Vis spectrophotometer (Perkin Elmer). The betacyanin content (BC) and betaxanthin content (BX) were calculated using the following equation:

B (mg/g) = (*A* × DF × Mw ×*V_d_*/ε*LW_d_*) (3)

where *A* is the absorption value at the absorption maximum of 535 and 483 nm for betacyanins and betaxanthins, respectively; DF is the dilution factor; *V_d_* is the dried pulp solution volume (mL); *W_d_* is the dried pulp weight (g); and L is the path length (1 cm) of the cuvette. For the quantification of the betacyanin and betaxanthin contents, we used the molecular weight (Mw) and molar extinction coefficient (ε) of betanin (Mw = 550 g/mol; ε = 60,000 L/mol cm in water; λ = 536 nm) and indicaxanthin (Mw = 308 g/mol; ε = 48,000 L/mol cm in water; λ = 481 nm), respectively. The measurements were performed in triplicate and the results were expressed as mg betacyanin/100 g extrudate and mg betaxanthin/100 g extrudate.

#### 3.5.8. Betacyanin Retention

The retention of betanin and isobetanin was determined using a method reported [52]. Briefly, 20 μL of extract were injected into Agilent Technologies Equipment 1200 Series Rapid Resolution LC system (Santa Clara, CA, USA) equipped with a quart pump, a reverse-phase Nucleosil column (C_18_ 250 × 4.6 mm; 0.5 μm particle size), and a UV detector. The mobile phase A was methanol/0.05 M KH_2_PO_4_ (18:82 v/v) adjusted to pH 2.75 using phosphoric acid, and the mobile phase B was methanol. The extracts were analyzed using a gradient that started with 100% A, reaching 80% A and 20% B in 20 min at a flow rate of 1 mL/min. Detection was performed at 536 nm. All determinations were performed in duplicate and reported as retention (%), which was calculated as (*X/X_0_*) *× 100*; where *X* is the area of the extrudate and *X_0_* is the area obtained for the starch/encapsulated mix before the extrusion-cooking process.

### 3.6. Calculation of the Kinetic Parameters

The degradation kinetic of betalains can be described by a general reaction rate expression [53]:

*ln* (*C_t_ − C_0_*) *= −kt* (4)

where *C_0_* is the initial component content, *C_t_* is the component content at the time *t*, *k* is the reaction rate constant, and *t* is the time. The temperature dependence of this constant (*k*) can be described by the Arrhenius equation:

*K =A_0_ exp* (−*E_a_/RT*) (5)

where *A_0_* is the frequency factor or pre-exponential constant, *E_a_* is the activation energy of the reaction, *R* is the universal gas constant, and *T* is the absolute temperature.

### 3.7. Data Analysis

The statistical design was a 4 × 3 completely randomized factorial design; extrusion temperature (80, 100, 120, and 140 °C) and screw speed (225, 275, and 325 rpm) were considered as independent variables. The properties of the extrudate were considered as dependent variables. The analyses of variance and the differences between means by Tukey’s test were performed using the software Minitab version 16 [54] at a significance level of 0.05. 

## 4. Conclusions 

The extrusion cooking process affected the retention of encapsulated red cactus pear pigments; however, the obtained retention values were higher than those reported in other extrusion studies. Higher screw speed and temperature result in a lower *tr_m_* of the material in the extruder. The results show that shorter exposure time of the components of interest to high temperature conditions, providing greater retention of total polyphenol, betacyanins, and betaxanthins, as well as antioxidant activity. The *k* values of betacyanins were higher than those of betaxanthins, whereas the activation energy for the degradation of betaxanthins was greater than that for the degradation of betacyanins; thus, betaxanthins are more susceptible to temperature increases during the extrusion cooking process. 

Despite the degradation of bioactive compounds contained in red cactus pear, the retention achieved by the extrusion cooking process suggests that encapsulated cactus pear powder is a promising natural pigment that can be used in extruded products. Furthermore, because it has antioxidant activity, it could have beneficial effects on consumers’ health.
